# Everolimus and Sirolimus in Combination with Cyclosporine Have Different Effects on Renal Metabolism in the Rat

**DOI:** 10.1371/journal.pone.0048063

**Published:** 2012-10-31

**Authors:** Rahul Bohra, Wenzel Schöning, Jelena Klawitter, Nina Brunner, Volker Schmitz, Touraj Shokati, Ryan Lawrence, Maria Fernanda Arbelaez, Björn Schniedewind, Uwe Christians, Jost Klawitter

**Affiliations:** 1 iC42 Clinical Research & Development, Department of Anesthesiology, University of Colorado Denver, Aurora, Colorado, United States of America; 2 Abteilung für Allgemein-, Viszeral- und Transplantationschirurgie, Charité, Berlin, Germany; Mario Negri Institute for Pharmacological Research and Azienda Ospedaliera Ospedali Riuniti di Bergamo, Italy

## Abstract

Enhancement of calcineurin inhibitor nephrotoxicity by sirolimus (SRL) is limiting the clinical use of this drug combination. We compared the dose-dependent effects of the structurally related everolimus (EVL) and sirolimus (SRL) alone, and in combination with cyclosporine (CsA), on the rat kidney. Lewis rats were treated by oral gavage for 28 days using a checkerboard dosing format (0, 3.0, 6.0 and 10.0 CsA and 0, 0.5, 1.5 and 3.0 mg/kg/day SRL or EVL, n = 4/dose combination). After 28 days, oxidative stress, energy charge, kidney histologies, glomerular filtration rates, and concentrations of the immunosuppressants were measured along with ^1^H-magnetic resonance spectroscopy (MRS) and gas chromatography- mass spectrometry profiles of cellular metabolites in urine. The combination of CsA with SRL led to higher urinary glucose concentrations and decreased levels of urinary Krebs cycle metabolites when compared to controls, suggesting that CsA+SRL negatively impacted proximal tubule metabolism. Unsupervised principal component analysis of MRS spectra distinguished unique urine metabolite patterns of rats treated with CsA+SRL from those treated with CsA+EVL and the controls. SRL, but not EVL blood concentrations were inversely correlated with urine Krebs cycle metabolite concentrations. Interestingly, the higher the EVL concentration, the closer urine metabolite patterns resembled those of controls, while in contrast, the combination of the highest doses of CsA+SRL showed the most significant differences in metabolite patterns. Surprisingly in this rat model, EVL and SRL in combination with CsA had different effects on kidney biochemistry, suggesting that further exploration of EVL in combination with low dose calcineurin inhibitors may be of potential benefit.

## Introduction

The calcineurin inhibitors cyclosporine (CsA) and tacrolimus form the basis of most immunosuppressive protocols early after organ transplantation to prevent graft rejection [1], [2]. Over the last three decades, calcineurin inhibitors have significantly improved short-term survival of transplant organs [3]. Recent analyses have also indicated an increase of renal allograft half-lives, albeit long-term results are still unacceptable [2]. Calcineurin inhibitor-related toxicity was identified as one of the main reasons for long-term failures. The most limiting side effects of calcineurin inhibitors are nephrotoxicity [4], [5], [6] and neurotoxicity [7], [8]. Other adverse effects, such as diabetes, hyperlipidemia and hypertension, are probably responsible for the high cardiovascular risk of transplant patients. While cardiovascular complications are the major cause of death in kidney transplant patients with functioning transplant [6], chronic renal allograft injury is the principal cause of late renal allograft loss after the first year post transplant [6], [9], [10], [11]. In an effort to prevent calcineurin inhibitor-induced nephrotoxicity, many studies detailing attempts to minimize or wean patients off of these medications have shown that improvement in renal function is often obtainable only with an increase in the incidence of alloimmune reactions. A retrospective analysis of 25,045 kidney transplant patients with good graft function indicated an association between withdrawing maintenance CsA or tacrolimus or reducing the dose of these agents below certain thresholds after the first year post-transplant, and an increased risk of graft loss [12]. Thus, developing calcineurin inhibitor-based long-term maintenance immunosuppressive drug regimens with improved long-term tolerability is a highly desirable endeavor.

**Figure 1:**
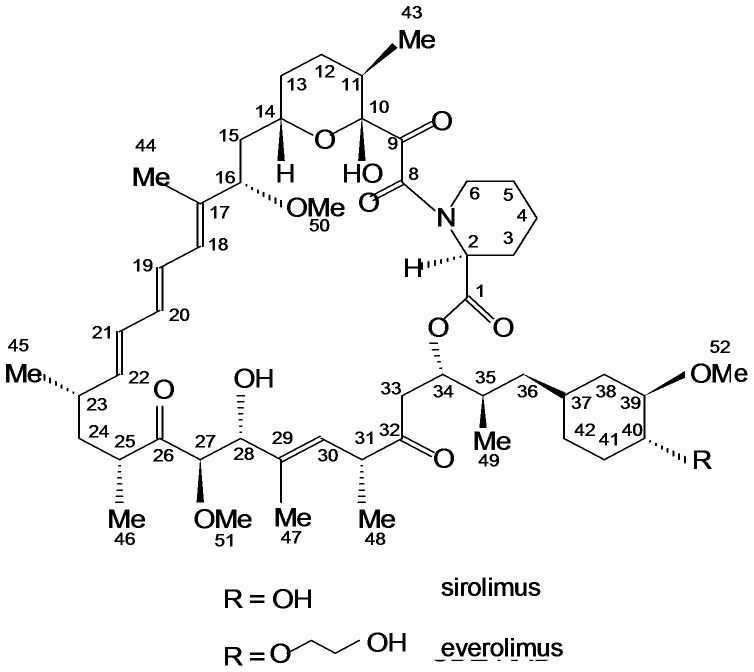
Structures of sirolimus and everolimus. Atom numbering follows the IUPAC (International Union of Pure and Applied Chemistry) nomenclature.

The primary problem when considering the use of calcineurin inhibitors is their low therapeutic index. One strategy to expand the therapeutic index of a calcineurin inhibitor-based immunosuppressive drug regimen is to combine immunosuppressive agents that interact in a synergistic fashion and allow for dose reduction of the combination partners, thus reducing toxicity while maintaining immunosuppressive potency [2]. Promising combination partners for calcineurin inhibitors are the structurally related inhibitors of the mammalian target of rapamycin (mTOR) sirolimus (SRL) and everolimus (EVL) (Figure 1), which both synergistically enhance immunosuppressive activity of calcineurin inhibitors [13], [14]. However, pivotal phase III-clinical studies found that when combined with full-dose CsA, these mTOR inhibitors actually have the potential to enhance CsA nephrotoxicity [15], [16], [17]. For SRL this was confirmed in mechanistic studies in the rat [18], [19].

As mentioned above, neurotoxicity is a clinically relevant problem associated with exposure to calcineurin inhibitors [7], [8]. In a series of studies we systematically assessed the effects of CsA on brain metabolism alone and in combination with mTOR inhibitors [20], [21], [22], [23], [24], [25]. These studies showed SRL to enhance and surprisingly EVL to antagonize the negative effects of CsA on energy metabolism in the brain. However, it was noticed that this only was the case if the CsA concentrations did not exceed the EVL concentrations by a factor of 5. At higher ratios, EVL behaved just like SRL.

The goal of the present study was to test the hypothesis derived from our previous observations in the brain that SRL, but not the structurally related EVL (Figure 1), enhances the negative effects of CsA on rat kidney metabolism.

This hypothesis was tested in an established rat nephrotoxicity model [18], [19] whereby kidney tissue and urine of treated rats were analyzed using a comprehensive biochemical profiling approach. A key feature of this rat nephrotoxicity model is that immunosuppressants were dosed so that ideally no histological changes occur during the observation period. Typically, histological damage goes parallel with secondary responses such as inflammation that *per se* already change cell and urine metabolite patterns. This means that they cause “metabolic noise” that makes it difficult, if not impossible, to discern which of the metabolic changes are specific for the study drugs. For this reason, we also decided not to use a salt-depleted rat model, a model that has extensively been used for studying immunosuppressant toxicity [26]. Salt-depleted rat models show accelerated development of immunosuppressant-induced histological changes, but as shown by our group, salt-depletion *per se* already leads to marked changes in kidney biochemistry [27] limiting usefulness of this model when studying the biochemical effects of immunosuppressants on the kidney.

## Materials and Methods

### Animal model and ethics statement

The study followed the protocol as described in [18], [19]. Male Lewis rats (10–14 weeks old, weighing 280–330 g, from Charles River Laboratories Wilmington, MA) were fed a normal salt diet and housed in temperature and light controlled cages with access to tap water and food *ad libitum*. Studies were conducted in accordance with the National Institutes of Health guidelines (NIH publication No. 80-123) once the study received approval by the University of Colorado Institutional Animal Care and Use Committee (Aurora, CO). Rats were weighed and examined daily and treatment was started following at least two weeks of acclimatization.

Drug treatment. Rats were treated with CsA, EVL, SRL or combinations of CsA+SRL and CsA+EVL. All drugs were administered by oral gavage at a constant volume (1 mL) in skim milk (1% fat) once daily. Animals were treated for 28 days. When combined, drugs were given simultaneously. Commercial oral formulations of CsA (Neoral, Novartis Pharma, East Hanover, NJ), SRL (Rapamune, Wyeth-Ayerst/Pfizer, Princeton, NJ), and EVL (Zortress, Novartis Pharma, East Hanover, NJ) were purchased from a local pharmacy.

### Experimental design

To assess the dose-dependency of drug effects on kidney biochemistry, a checkerboard dosing scheme was used (Table 1). A total of 128 rats were assigned to the following study groups: controls (vehicle treatment: 1 mL skim milk, n = 8; all other groups n = 4), groups treated with 0, 3.0, 6.0, 10.0 mg CsA/kg/d or 0, 0.5, 1.5, 3.0 mg SRL or EVL/kg/d or the following combinations: CsA+SRL (3.0, 6.0, 10.0 mg CsA/kg/d and 0.5, 1.5, 3.0 mg SRL/kg/d, every possible combination) and CsA+EVL (3.0, 6.0, 10.0 mg CsA/kg/d and 0.5, 1.5, 3.0 mg EVL/kg/d, every possible combination). Every week, a blood sample (1 mL total) was drawn under mild isoflurane anesthesia from the jugular vein into a tube containing the anti-coagulant ethylenediaminetetraacetic acid (EDTA) to measure trough blood concentrations ensuring that the rats had been exposed to the study drugs. Rats were placed in metabolic cages for 24 hours on study days, 0 (pre-dose day, baseline), 7, 14, 21 and 27 (after the penultimate dose) for urine collection. The urine samples were used for metabolic profiling and the measurement of 15-F_2t_-isoprostane concentrations. Rats were sacrificed on the morning of study day 28, 4 hours after the last study drug dose. Glomerular filtration rates were directly assessed (*vide infra*). Hereafter, the following samples were collected: EDTA blood sample, one kidney was freeze-clamped while still perfused as previously described [18], [28] and used for the measurement of high-energy phosphates. The other kidney was used for histology.

**Table 1:** Overview of study groups.

		Sirolimus [mg/kg/day]						Everolimus [mg/kg/day]			
**CsA [mg/kg/day]**		0	0.5	1.5	3	**CsA [mg/kg/day]**		0	0.5	1.5	3
	0	4	4	4	4		0	4	4	4	4
	3	4	4	4	4		3	4	4	4	4
	6	4	4	4	4		6	4	4	4	4
	10	4	4	4	4		10	4	4	4	4

Due to the critical impact of the ratios of sirolimus/cyclosporine and everolimus/cyclosporine on tissue metabolism as observed in previous studies, a checkerboard dosing scheme was used. Four rats were assigned to each group. All possible cyclosporine/sirolimus and cyclosporine/everolimus combinations of the following doses were tested: cyclosporine: 3.0, 6.0, 10.0 mg/kg/day; sirolimus and everolimus: 0.5, 1.5, 3.0 mg/kg/day. The “0.0 mg/kg/day” groups were given 1 mL vehicle solution (skim milk) instead of the active drug. “0.0/0.0 mg/kg/day” groups served as the vehicle controls.

### Measurements

#### Glomerular filtration rates

Renal function was determined using the fluorescein isothiocyanate (FITC)-inulin method [29], [30]. In brief: glomerular filtration rates (µL/min) were calculated using the formula (UxV/P) where U = inulin concentration in urine, V = urine output over time and P = inulin concentration in plasma. For baseline corrections, blank control plasma and urine samples were loaded with different concentrations of inulin and fluorescence absorption was measured [18].

#### Quantification of immunosuppressants in EDTA whole blood and kidney tissues

All drug concentrations were determined 4 hours after the last dose on day 28 using a validated LC-MS assay [31]. In brief, whole blood samples (500 µL) were collected in EDTA tubes. Flash-frozen renal tissue (100 to 200 mg) was mortared in liquid nitrogen and homogenized with 2 mL KH_2_PO_4_ buffer (pH 7.4). For protein precipitation, 800 µL methanol and 0.2 mmol/L ZnSO_4_ (80/20, v/v) were added to 200 µL of blood or tissue suspension. Cyclosporin D (250 µg/L, Novartis Pharma, Basel, Switzerland) and zotarolimus (40 µg/L, Toronto Research Chemicals, North York, ON) were added as internal standards for CsA and EVR/SRL, respectively. After centrifugation (13,000 *g*, 5 min, 4°C), 100 µL of the supernatant was injected onto the extraction column of the LC-MS/MS system. Key method performance parameters are described in [31].

#### Histology

For hematoxylin and eosin (H.E.) staining, kidney tissue samples were fixed in 10% buffered formaldehyde and embedded in paraffin, incubated for 5 minutes in Harris hematoxylin solution and then for 60 seconds in eosin solution. Sections were washed with plain water, differentiated in 1% hydrochloric acid +50% ethanol, and the stain intensity was optimized in ammonia water. Finally, sections were rinsed in 70% ethyl alcohol and dehydrated in xylene solution.

Evaluation of kidney histology was carried out in a blinded manner using a semi-quantitative scoring system. Histologies were scored with regards to their tubular epithelial aspects, glomerular and vascular alterations in accordance with the modified Banff classification criteria [32]. Digitized images ranging from 10×5 to 10×20 magnification were acquired using a Zeiss microscope fitted with a Zeiss light video camera (Carl Zeiss, Maple Grove, MN) alongside a computer with Adobe PhotoShop CS5 software (Adobe, San José, CA). In 20 randomly selected non-overlapping fields per rat on H.E. and Periodic Acid Schiff stains, tubular injury was graded (0 to 3) based on the presence of tubular atrophy ( = interstitial widening) and the presence/degree of isometric tubular vacuolization: 0 = no changes present, grade 1 = ≤25%; grade 2 = 26 to 50% and grade 3≥50% tubular injury involvement. Interstitial fibrosis was scored as a sign of architectural destruction: no changes (Grade 0), grade 1 = 1–25%; grade 2 = 26–50% and grade 3≥50% interstitial fibrosis. Glomerular injury was graded 0–3 for sclerosis (n = 50 tubules) and mesangial matrix expansion (n = 50) as a marker for glomerular ischemia and damage. Renal arterioli were evaluated with respect to the presence of hyalinosis or sclerosis. Grade 0 = no arteriolar changes; mild-moderate (grade 1) = 1 arteriole affected; moderate-severe (grade 2) = 1–2 arterioles affected; severe (grade 3) = more than 2 arterioles affected.

#### ^1^H-nuclear magnetic resonance spectroscopy (MRS)-based metabolic profiling in urine

^1^H-MRS urine analysis was performed using a Varian INOVA NMR 500 MHz spectrometer. Five hundred and fifty µL of urine was buffered with 73 µL of 0.2 mol/L K_2_HPO_4_ buffer in D_2_O prior to analysis. The final pH was adjusted to 5.65–5.75 with NaOD and DCl. To suppress water in urine, a standard Varian pre-saturation sequence was used. ^1^H-MRS spectra were recorded at 500 MHz using a spectral width of 12 ppm creating 32 K data arrays and 64 scans with 90° flip angle using relaxation time of 14.8 seconds. The 1D WINNMR and TOPSPIN software (Bruker, Karlsruhe, Germany) were used for MRS data processing. Spectra were calibrated in reference to the creatinine signal at 3.05 ppm and were corrected for phase, baseline distortions and normalized to the total integral of the MRS spectra.

#### Quantification of 15-F_2t_-isoprostane (8-iso-prostaglandin-F2α) in urine

15-F_2t_-isoprostane is a marker for oxidative stress [33]. Increasing oxygen radical concentrations has been shown to mediate CsA toxicity [20]. Thus, 15-F_2t_-isoprostane concentrations in urine are a marker that seems directly linked to CsA's toxicodynamic mechanism [34]. Urine samples were analyzed using a validated LC-MS/MS method [35], [36].

#### Quantification of high-energy phosphate concentrations in kidney tissues

Nucleotide energy phosphates were measured using a validated LC-MS assay after extraction of kidney tissues with perchloric acid [28]. The energy charge was calculated as [(ATP+0.5*ADP)/(ATP+ADP+AMP)].

### Data analysis and statistical procedures

For data analysis, a combination of targeted/hypothesis-driven approach (analysis of variance (ANOVA), correlation analysis) and a non-targeted/unsupervised approach based on principal components analysis (PCA) was used. All numerical data are presented as means± standard deviation. A one-way ANOVA followed by least significant difference (LSD) or Tukey *post hoc* analysis, was used to determine differences among groups. Significance levels were set at p<0.05 for all tests. The following software packages were used for data analysis: SigmaPlot (version 11.0, Systat Software, Point Richmond, CA); and IBM SPSS statistics (version 20.0, IBM/SPSS, Chicago, IL). In terms of the PCA, data buckets were formed in 0.04 ppm intervals and PCA was performed using the AMIX software (Bruker, Rheinstetten, Germany). The MRS water signal region was excluded from analysis.

## Results

### Blood drug and kidney tissue concentrations (Figure 2)

**Figure 2:**
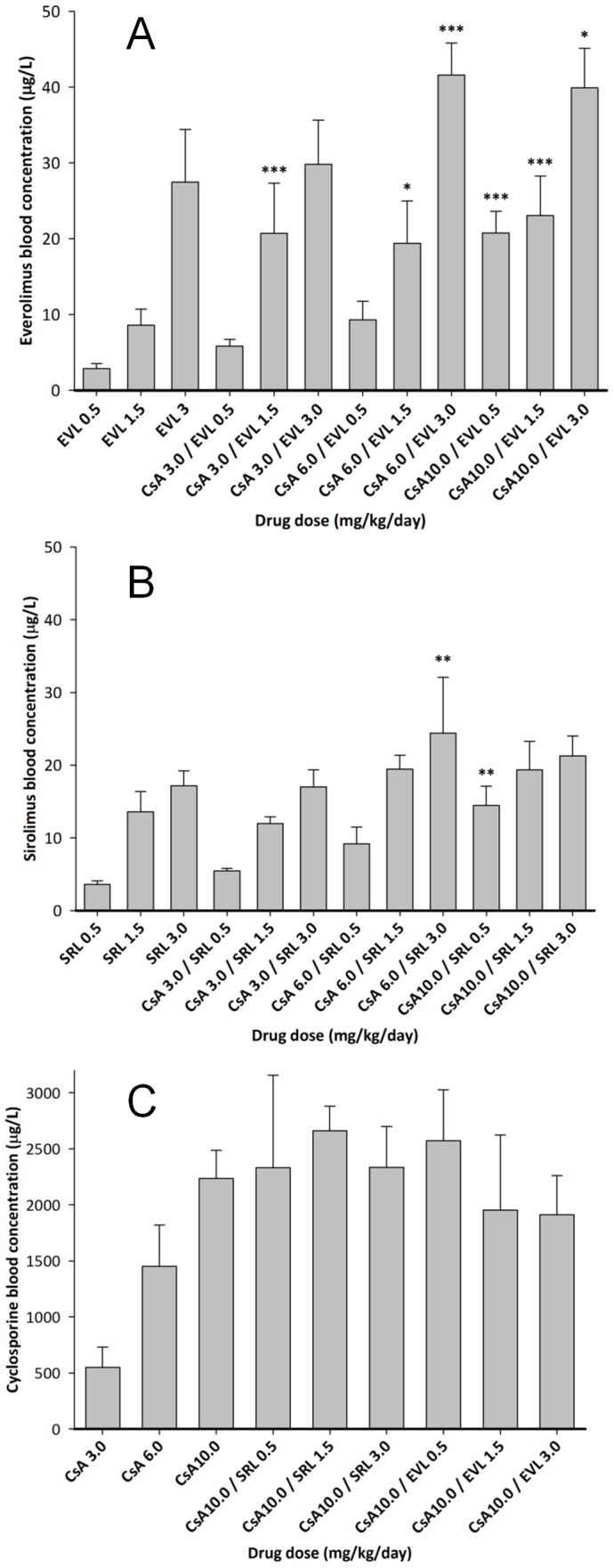
Blood concentrations of everolimus (EVL) (A), sirolimus (SRL) (B) and cyclosporine (CsA) (C) in selected study groups. EDTA whole blood samples were collected after 28 days of treatment and 4 hours after the last dose. Concentrations of the study drugs were measured using a validated, specific LC-MS assays [31]. Data among groups was compared using analysis of variance in combination with Tukey's post-hoc test. Significance levels: * = p<0.05, ** = p<0.01, *** = p<0.001. Only significances between groups receiving a combination of cyclosporine and sirolimus or cyclosporine and everolimus compared to the concentrations in the blood of rats receiving the corresponding dose alone are shown. The numbers in the x-axis labels give the doses in mg/kg/d. Thus, for example, CsA 10.0/SRL 3.0 means that this group of rats was treated with a combination of 10 mg/kg/day cyclosporine and 3.0 mg/kg/day sirolimus for 28 days.

As expected, there was a marked drug-drug interaction between EVL and CsA. For example, when 0.5 mg EVL/kg/day was co-administered with CsA (Figure 2A), the blood concentration of EVL was 2.9±0.66 µg/L, 5.8±0.92 µg/L, 9.3±2.4 µg/L and 20.7±2.9 µg/L when dosed alone and with 3, 6 and 10 mg CsA/kg/day, respectively. A similar drug-drug interaction was observed with SRL. The corresponding SRL concentrations after 0.5 mg/kg/day for 28 days were 3.6±0.50 µg/L without CsA and 5.4±0.35 µg/L, 9.2±2.3 µg/L and 14.5±2.7 µg/L when co-administered with 3, 6 and 10 mg CsA/kg/day (Figure 2B). However, neither co-administration of EVL nor SRL affected CsA blood concentrations (Figure 2C). In a similar fashion, CsA increased kidney tissue concentrations of SRL and EVL, while SRL and EVL co-administration seemed to reduce CsA distribution into the kidney (Table S1). This drug interaction seemed to affect EVL more than SRL resulting in higher EVL tissue concentrations in combination with CsA when compared to those after corresponding SRL doses.

### 15-F_2t_-isoprostane concentrations in urine (Figure 3)

**Figure 3:**
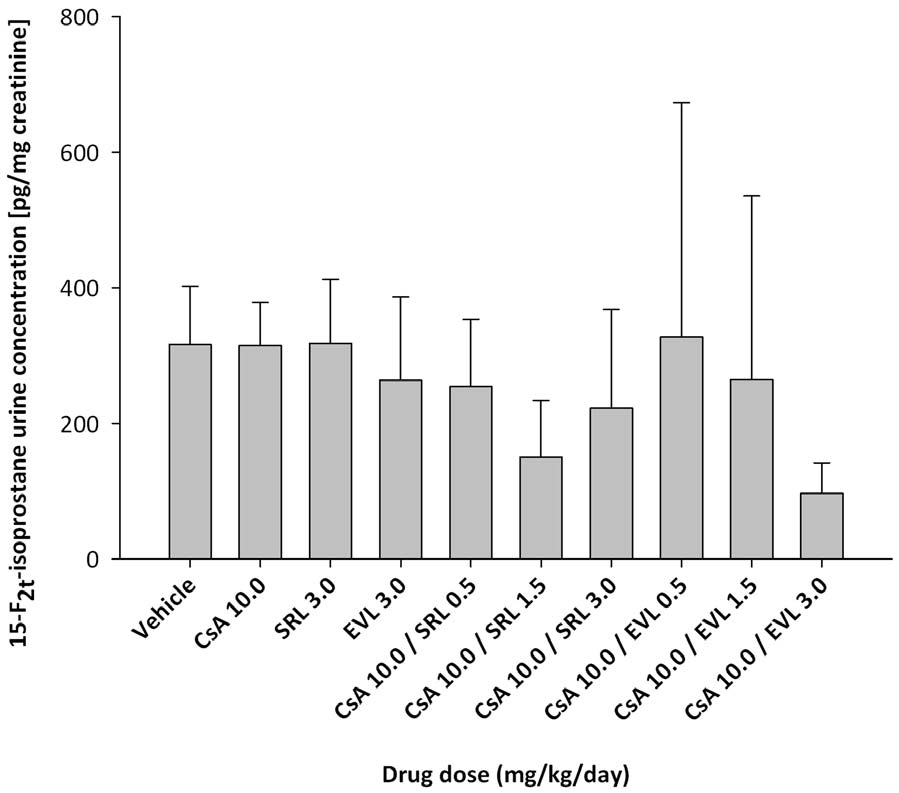
15-F_2t_-Isoprostane concentrations in urine after 28 days of treatment with cyclosporine (CsA), sirolimus (SRL) and everolimus (EVL) alone or in combination with cyclosporine (10 mg/kg/day). 15-F_2t_-isoprostane concentrations were measured using a validated, highly sensitive and specific LC-MS/MS assay [35], [36]. To compensate for differences in the concentration of urine samples, isoprostane concentrations were normalized based urine creatinine concentrations. All bars are means ± standard deviations (n = 4). Data among groups was compared using analysis of variance in combination with Tukey's *post-hoc* test and no significant differences were found. The numbers in the x-axis labels give the doses in mg/kg/day.

As assessed using ANOVA there were no statistically significant differences in concentrations of 15-F_2t_-isoprostane in urine between any of the treatments and controls.

### High-energy phosphates in kidney tissues (Figure 4)

**Figure 4:**
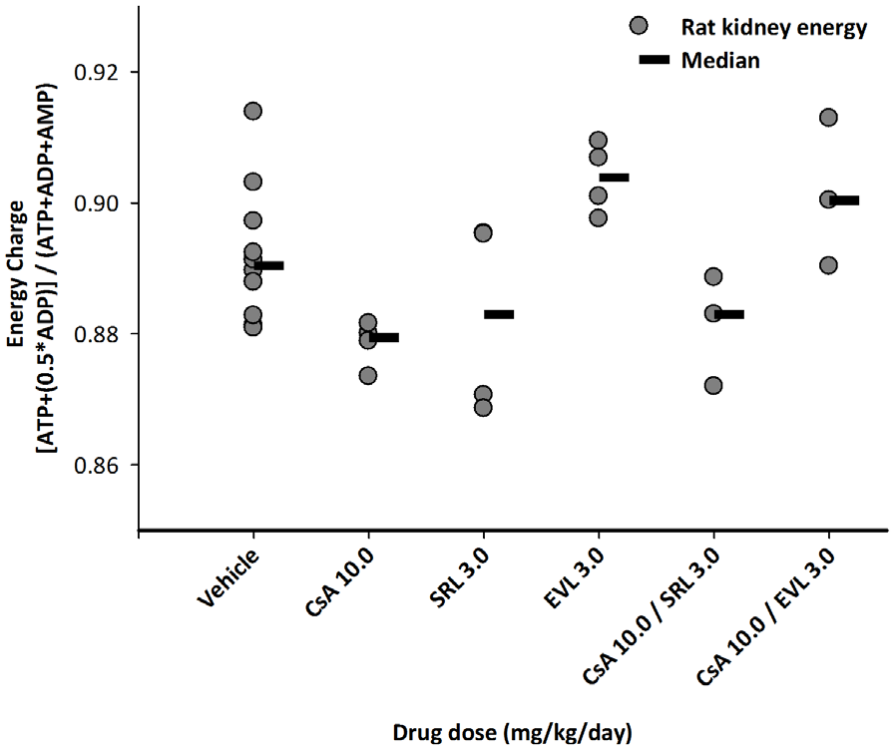
The effects of cyclosporine (CsA), sirolimus (SRL) and everolimus (EVL) alone or in combination with cyclosporine (10 mg/kg/day) on the energy charge in kidney tissues after 28 days of exposure. The rat kidneys were freeze clamped while still perfused. This was critical to avoid any changes in high-energy phosphate concentrations during tissue collection [28]. Individual data points are shown and the horizontal lines show the median for each group. Please note that in some cases data points overlap and appear as one. Data among groups was compared using analysis of variance and no statistically significant differences were found. The numbers in the x-axis labels give the doses in mg/kg/day.

When compared to the energy charge in kidney tissues from the control group (0.890±0.01, mean ± standard deviation) no statistically significant differences were found, as assessed using ANOVA.

### Glomerular filtration rate (GFR) (Figure 5)

**Figure 5:**
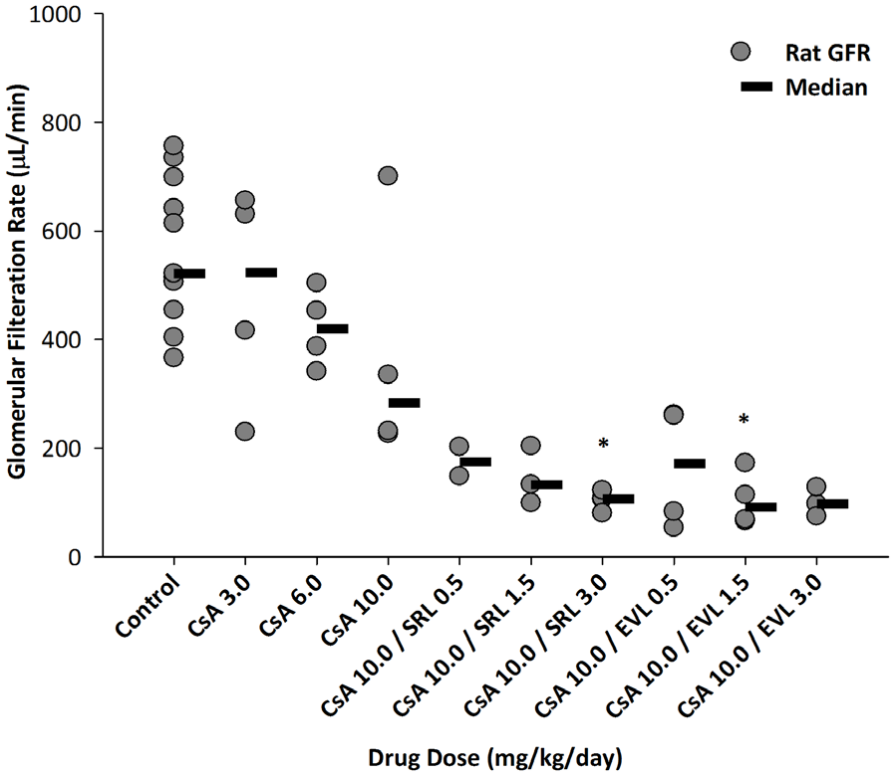
The effects of cyclosporine (CsA), sirolimus (SRL) and everolimus (EVL) alone or in combination with 10 mg/kg/d cyclosporine on glomerular filtration rates (GFR). GFRs were directly measured using the fluorescein isothiocyanate (FITC)-inulin method [29], [30]. All GFR values were corrected for animal body weight. Since the GFRs in some of the rats could not be determined due to technical difficulties, the numbers of observations in some groups is less than 4. Therefore, individual results are shown. The horizontal lines show the median of each group. Please note that in some cases data points overlap and may appear as one. The numbers in the x-axis labels give the doses in mg/kg/d. *: p<0.05 (compared to control, analysis of variance in combination with Tukey's *post-hoc* test).

As expected, the GFRs declined with higher CsA doses: vehicle controls: 545.9±142.2 (µL/min, mean ± standard deviation); 483±200 (3 mg/kg/d); 421±72 (5 mg/kg/day); and 373±224 (10 mg/kg/day). When 10 mg/kg/day CsA was combined with SRL and EVL, there was an additional effect on the GFRs when compared to using CsA alone (10 mg/kg/day). In case of combination with 3 mg/kg/day of SRL and EVL the GFR was decreased to 104±18 and 100±27, respectively.

### Histology (Figure S1 and Table S2)

As expected and intended, overall histological damage was mild. Histology revealed no difference in injury scores between SRL or EVL in combination with CsA (Figure S1). Although the data showed inter-individual variability, it appeared that the histology injury scores were mostly driven by the CsA dose. However, at the highest CsA dose (10 mg/kg/day) the addition of SRL or EVL showed the tendency to enhance the effect of CsA although this effect did not reach statistical significance.

### Metabolite concentrations in urine (Table 2, Figures 6, 7, 8, Figures S2, S3 and S4)

**Figure 6:**
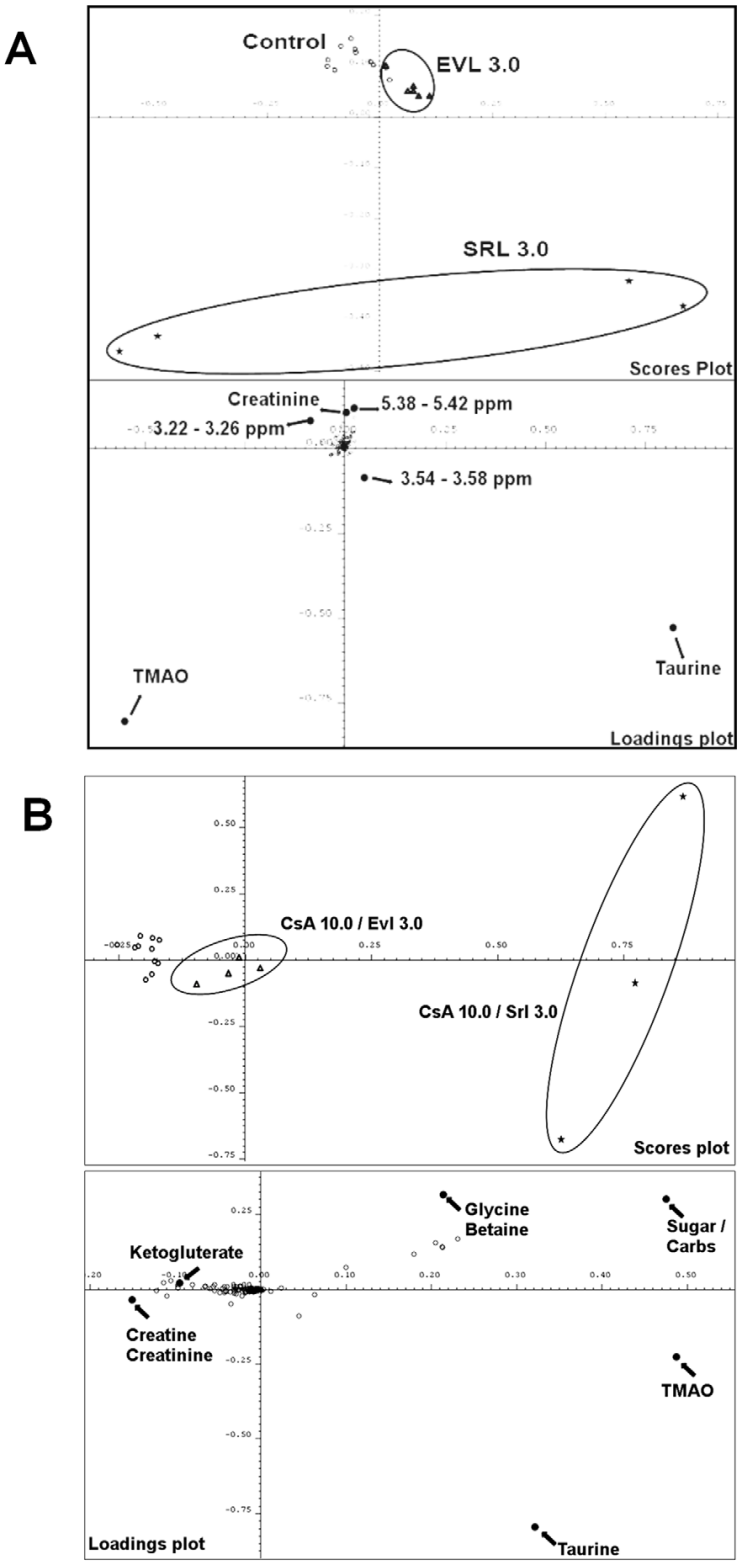
Principal components analysis of rat urine ^1^H-MRS spectra after 28 days of drug treatment (A) based on spectra recorded in urine from rats treated with 3 mg/kg/day EVL and 3 mg/kg/day SRL alone as well as the vehicle controls and (B) from rats treated with combinations of CsA (10 mg/kg/day) with EVL or SRL (both 3 mg/kg/day). The spectra were reduced to ppm buckets (binning), which represent the area-under-the-curve in a certain spectral region. This created an ensemble of XY-tables (spectral region *versus* integral), the so-called bucket tables. The spectra were analyzed using a Principal Components Analysis (PCA) (top) and Partial Least Squares (PLS) fit analysis (bottom; AMIX software, Bruker, Rheinstetten, Germany). In the PCA, the principal components are constructed in such a way that the first explains most of the variance in the ensemble, the second explains the second most, and so on. The clustering analysis of the scores plots, the principal component 1 *versus* the principal component 2, was used to determine whether groups of spectra differed from each other. Thus, hidden phenomena that were not obvious from the usual spectral dimension could be discovered. The spectral regions that caused the separation were identified in the loading plots, which form the link back to the spectral dimension.

**Figure 7:**
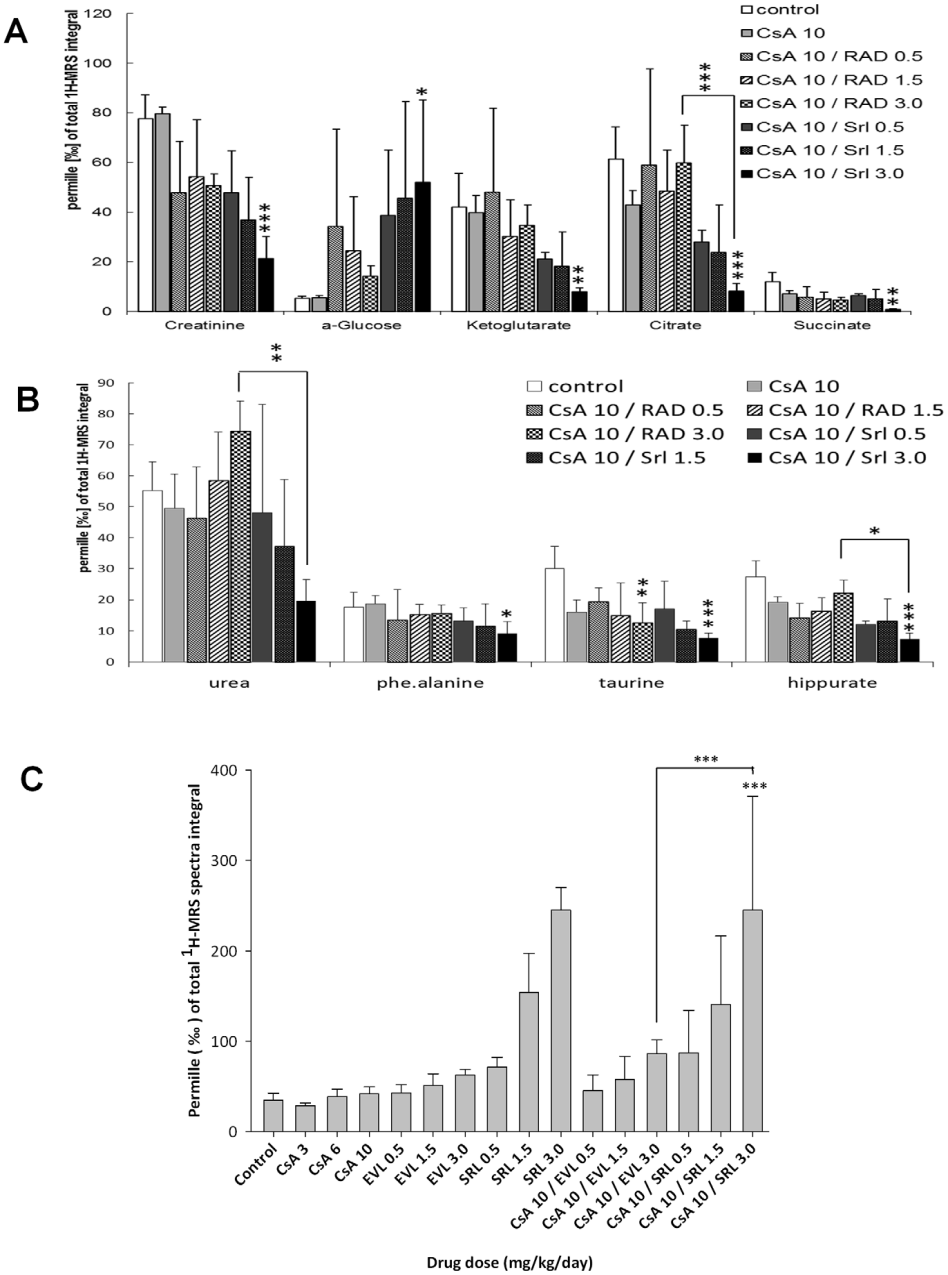
Comparison of the effects of sirolimus (SRL) and everolimus (EVL) in combination with cyclosporine (CsA, 10 mg/kg/day) on urine metabolite patterns after 28 days of exposure as assessed using ^1^H-MRS. (A) Comparison of the effects on creatinine, α-glucose, citrate, ketoglutarate and succinate concentrations in urine; (B) comparison of the effects on hippurate, urea, phenylalanine and taurine concentrations in urine; (C) comparison of the effects on trimethylamine oxide (TMAO) concentrations in urine. Values were normalized based on the total spectrum integral to compensate for differences in urine concentrations. Thus, all values are permille of the total integral and are reported as means ± standard deviations (n = 4). Data among groups was compared using analysis of variance in combination with Tukey's *post-hoc* test. Significance levels: * = p<0.05; ** = p<0.01; *** = p<0.001. The numbers in the legends give the doses in mg/kg/day. In terms of the effects of the study drugs on urine metabolite patterns when dosed alone, please refer to Table 2.

**Figure 8:**
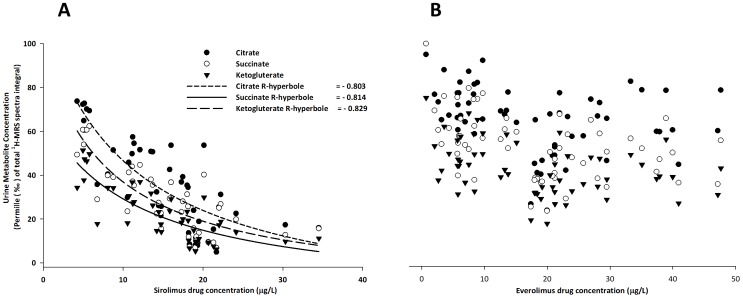
Regression analyses of sirolimus (SRL, A) and everolimus (EVL, B) blood concentrations and relative Krebs cycle metabolite concentrations in urine. Concentrations of citrate, ketoglutarate and succinate as measured using ^1^H-MRS were normalized based on the total spectrum integral to compensate for differences in urine concentrations. Thus, all values are reported as permille of the total integral. The analysis is based on SRL (n = 44) and EVL (n = 59) observations.

**Table 2:** Comparison of the effects of cyclosporine (10 mg/kg/day), sirolimus (3 mg/kg/day) and everolimus (3 mg/kg/day) on key urine metabolites after 28 days of exposure.

	Vehicle Controls	Cyclosporine	Sirolimus	Everolimus
***Hippurate***	27.5±5.2	19.2±1.8	17.6±2.2	28.4±3.8
***Creatinine***	77.7±9.6	79.7±2.5	33.7±4.6***	59.5±6.1
***α-Glucose***	5.3±0.84	5.5±0.84	3.0±3.1	3.6±0.80
***Succinate***	11.9±3.9	7.1±1.3	5.8±0.97	11.8±2.9
***Citrate***	61.3±13.0	42.8±5.8	32.4±6.4	65.8±9.3
***Urea***	55.2±9.3	49.5±11.1	20.4±8.5	71.5±14.6
***TMAO***	34.7±7.8	41.9±7.2	245.1±24.8***	62.5±6.6
***Taurine***	30.2±7.1	16.1±3.7*	10.6±2.5***	20.4±5.2
***Ketoglutarate***	41.9±13.7	39.7±7.1	17.2±2.9*	42.5±10.4

Urine metabolites were measured using ^1^H-MRS. Values were normalized based on the total ^1^H-MRS spectrum integral to compensate for differences in urine concentration. Thus, all values are permille of the total integral and are reported as means ± standard deviations (n = 4). Data among groups was compared using analysis of variance in combination with Tukey's *post-hoc* test. Significance levels:

* = p<0.05;

** = p<0.01;

*** = p<0.001 (all in comparison to vehicle controls).

Urine metabolite patterns were assessed using ^1^H-MRS. Representative ^1^H-MRS spectra are shown in Figure S2. An unsupervised PCA was conducted in order to obtain first information as to whether SRL or EVL in combination with CsA have different effects on the kidney (Figure 6). Instead of comparing selected urine metabolites, the PCA was based on the complete urine MRS spectra. MRS spectra of SRL-treated rats showed clear separation from controls and EVL-treated groups when treated with single drugs (Figure 6a) and in combination with CsA (Figure 6b). The different effects of CsA+SRL versus CSA+EVL were also confirmed by a gas chromatography- mass spectrometry (GC-MS) based assay (Figures S3 and S4). The MRS spectral regions that caused the separation were identified in the loading plots, forming a link back to the spectral dimension. As indicated by the loading plots, among other metabolites, trimethylamine oxide (TMAO) and taurine concentrations in urine contributed to the separation of groups treated with EVL or SRL with or without CsA (Figure 6).

Interestingly, the PCA shown in Figure 6A indicated that urine metabolite patterns were already differentially affected by SRL and EVL when dosed without CsA. As PCA can only be a first test and is prone to confounders, we assessed the differences of specific urine metabolite concentrations using analysis of variance in combination with Tukey's post-hoc test (Table 2, Figure 7). This analysis confirmed the results of the PCA. SRL (3 mg/kg/day) seemed to have a more extensive effect on urine metabolite patterns than EVL (3 mg/kg/day). In fact, when Krebs cycle metabolites, which are surrogate markers of proximal tubule mitochondrial function, were compared after 28 days of exposure to 3 mg/kg/day SRL or EVL, the following differences were observed: citrate: SRL *versus* EVL: 32.4±6.4 *versus* 65.8±9.3‰ of the total MRS integral (all mean ± standard deviation, n = 4), succinate: 5.8±1.0 *versus* 11.8±2.9, and ketoglutarate: 17.2±2.9 *versus* 42.5±10.4. There was also a difference in TMAO concentrations: SRL: 245.1±24.8 *versus* EVL: 62.5±6.6 (p<0.001). In contrast to SRL, the effects of EVL (3 mg/kg/day) on Krebs cycle metabolite and TMAO concentrations did not seem different from those of the vehicle controls (Table 2).

The effects of SRL and EVL on urine metabolite patterns were also different, when SRL or EVL were co-administered with 10 mg/kg/day CsA. Previous work using a similar rat model found CsA to mainly target the proximal tubule markedly increasing relative glucose and reducing Krebs cycle metabolite concentrations [18], [19]. These CsA effects were further enhanced by co-administration of SRL. The present study confirmed these results. The combination of CsA (10 mg/kg/day) with different SRL doses (0.5, 1.5 and 3.0 mg/kg/day) led to significantly increased relative glucose concentrations in urine. Interestingly, everolimus dose-dependently improved the effect on relative urine glucose concentrations. In fact, urine glucose concentrations at the highest EVL dose (combination of 10 mg/kg/day CsA with 3.0 mg/kg/day EVL) were similar to the vehicle controls yet different compared to those at the corresponding CsA+SRL doses (p<0.05, analysis of variance in combination with Tukey's *post-hoc* test, Figure 7A). Also, no notable differences existed between any of the EVL combinations with 10 mg/kg/day CsA and the vehicle controls when comparing Krebs cycle metabolite concentrations in urine. In contrast, most of the SRL and 10 mg/kg/day CsA combinations resulted in marked differences compared to the vehicle controls (citrate: p<0.0001, succinate; p<0.01, α-ketoglutarate: p<0.01, analysis of variance in combination with Tukey's *post-hoc* test, Figure 7A). Similar differences were observed, when comparing the effects of SRL and EVL in combination with 10 mg/kg/day CsA on hippurate (p<0.001, analysis of variance in combination with Tukey's *post-hoc* test, Figure 7B). TMAO is a cell metabolite present in the proximal tubule at relatively high concentrations and is an established injury marker of proximal tubule injury [37]. Figure 7C compares the TMAO concentrations in urine among samples collected after 28 days from different treatment groups. In this rat model when administered alone, SRL already increases TMAO concentrations compared to the vehicle controls (p<0.01, analysis of variance in combination with Tukey's *post-hoc* test). In contrast, CsA and EVL dosed alone did not affect urine TMAO concentrations at any of the tested doses. When combined with CsA (10 mg/kg/d), EVL had significantly less of an effect on TMAO urine concentrations than the same SRL dose (p<0.001, analysis of variance in combination with Tukey's *post-hoc* test, Figure 7C).

Moreover, potential correlations between immunosuppressant blood concentrations and their effects on the kidney were analyzed as shown in Figure 8. This analysis further confirmed the aforementioned observations that SRL blood concentration-dependently decreased relative Krebs cycle metabolite concentrations in urine (correlation coefficients (r): citrate: r = −0.80, ketoglutarate: r = −0.81, succinate: r = −0.83, n = 44) (Figure 8A), while there was no such correlation with EVL blood concentrations (n = 59) (Figure 8B).

## Discussion

One of the major challenges in studying the toxicity of calcineurin inhibitors such as cyclosporine and tacrolimus has been the lack of a suitable animal model. As assessed by histological changes, rats are known to be more resistant to calcineurin inhibitor toxicity when compared to humans [38]. As a proximal fluid, urine is in constant direct exchange with the kidney. Thus changes in kidney function and cellular metabolism are reflected in urine before detectable histological injury occurs [39]. The rat model used here and described in [18] assesses the effects of immunosuppressants and their combinations in the kidney either before histological changes occur or while only mild histological changes were observed. Thus, as expected, the histological changes observed in the normal fed rats in the present study were much less pronounced than those observed in other studies where rats were fed a low salt diet [40].

In a verification study, many of the changes in urine metabolite patterns observed in this model could mechanistically be linked to the effects of calcineurin inhibitors on protein expression and cell metabolism in the kidney [18]. In a further step, the changes in urine metabolite patterns caused by CsA observed in the rat model [18], [19] could also be translated into humans [34]. In this study, when compared to administration of an appropriate placebo, a single oral 5 mg/kg CsA dose resulted in changes of 15-F_2t_-isoprostane, citrate, hippurate, lactate, TMAO, creatinine and phenylalanine concentrations in urine from healthy individuals within 4 hours.

The doses of SRL and EVL administered in the present study were approximately 10-fold higher than those typically used in transplant patients. It is important to note that the drugs were dosed orally and that oral bioavailability of these drugs in rats is markedly lower than in humans. The CsA and SRL doses tested in the present study were based on our previous studies [18], [19] and were known to result in blood concentrations within or close to target blood concentrations maintained in transplant patients. EVL doses were chosen to match the SRL doses. Also, it is important to take into account that the blood concentrations shown in Figure 2 are not trough blood concentrations as are typically monitored in patients but concentrations in blood samples drawn 4 hours after dosing. This was due to the fact that rats were sacrificed 4 hours after the last dose to stay consistent with references [18], [19]. The blood concentrations in the present study ranged from 5.8±0.92 to 20.7±2.9 µg/L (all means ± standard deviations) for EVL and from 5.4±0.35 µg/L to 14.5±2.7 µg/L for SRL, both in combination with CsA, and thus spanned the clinical ranges recommended for maintenance kidney transplant patients in combination with CsA: 3.0 to 8.0 µg/L for EVL and 4.0 to 15.0 µg/L for SRL (trough blood concentrations, [41], [42]). CsA blood concentrations ranged from 551±179 µg/L (3 mg/kg/d) to 2235±251 µg/L (10 mg/kg/d) which were in the range of concentrations observed in transplant patients 4 hours after dosing [43]. The observed drug-drug interactions leading to higher SRL and EVL blood concentrations when combined with CsA than when dosed alone were expected as all three drugs are cytochrome P4503A and p-glycoprotein substrates in the small intestine and liver [44]. All study drugs are well-established substrates of active drug transporters such as P-glycoprotein and of cytochrome P450 drug metabolizing enzymes, most notably cytochrome P4503A, in the liver and small intestine [45], [46], [47], [48]. Thus, competitive interactions at these drug transporters and drug metabolizing enzymes are most likely involved in the observed pharmacokinetic interactions.

As aforementioned, our previous studies had indicated that the toxicodynamic interactions of CsA, SRL and EVL were dependent on the ratio of CsA/SRL and CsA/EVL exposure. In these studies it was observed that EVL antagonized some of the negative effects of CsA on cell metabolism in brain tissues and neuronal cells at concentration ratios of EVL: CsA ranging from 1∶3.3 to 1∶5 [22], [23], [24], [25]. As the best CsA/EVL concentration ratios in terms of avoiding toxicodynamic effects on rat kidneys were un known, it was necessary to study different dose CsA/SRL and CsA/EVL dose combinations and to implement the checkerboard dosing matrix shown in Table 1.

Changes in urine metabolite patters were the primary focus of the present study and, as intended, histology scores indeed indicated only mild changes and, however, did not discriminate among treatment groups. The present study included the direct measurement of GFRs as a functional parameter. GFRs were significantly changed by the study drugs indicating that CsA exposure was sufficient to cause functional changes in the kidney. Based on a salt-depleted rat model, Podder et al. observed an enhancement of the negative effects of CsA on GFRs when CsA was added [48]. In normal fed rats in the present study, we observed a trend towards lower GFRs when CsA was combined with the mTOR inhibitors, however, this effect was not statistically significant.

As aforementioned, it is well established that the increased formation of oxygen radicals plays a key role in CsA toxicity [20]. Interestingly, no increase of 15-F_2t_-isoprostane concentrations (normalized based on urine creatinine concentrations, compared to the vehicle control) was observed in any of the groups after 28 days of treatment. It seems reasonable to expect that in the present study after 28 days of exposure to the study drug regimens compensatory mechanisms to reduce oxidative stress, and thus reducing 15-F_2t_-isoprostane concentrations, may have been activated.

In the present study, SRL alone already affected urine metabolite patterns (Table 2, Figures 6A and 7C). This was also observed in previous studies [18], [19]. While these results suggested a direct or indirect negative effect of SRL on mitochondrial metabolism in the proximal tubule, the structurally related EVL did not have such an effect on kidney cell metabolism when dosed alone (Table 2). The differential effects of SRL and EVL on kidney metabolism became even more evident when both drugs were co-administered with CsA for 28 days. While SRL enhanced the negative effects of CsA on kidney metabolism confirming previous results in the rat [18], [19], many of the key metabolite concentrations in urine were similar to those in the urine from the control rats when CsA and EVL were combined (Figure 7). For most metabolites, there were significant differences after 28 days of CsA+SRL *versus* CsA+EVL treatment. It was interesting to note that glucose concentrations in urine (Figure 7A) were closer to those of the vehicle controls the higher the EVL doses.

The urinary concentration of creatinine, urea and ketoglutarate, dose-dependently decreased in the CSA+SRL group whereas in the CSA+EVL it remained basically unchanged. While the creatinine urinary concentration may potentially be influenced by the creatinine clearance and creatine catabolism, urea and ketoglutarate are involved and contribute to the urea cycle [49]. Creatinine synthesis and urea cycle are connected *via* the activity of the arginine∶glycine amindinotransferase (AGAT), which is mainly active in the kidney and pancreas. The enzyme produces ornithine and guanidine acetate, which are utilized in the urea cycle and for creatine synthesis in the liver [50]. The end products of urea cycle and creatine production are urea and creatinine, which are excreted *via* the kidney. There are two alternative explanations for the decrease of urea and creatinine concentrations in urine: a difference in the glomerular filtration rate could result in decreased excretion of urea and creatinine and drug-induced inhibition of AGAT. However, changes in GFR of rats receiving CSA 10.0+SRL 3.0 and CSA10+EVL 3.0 were similar. Indeed, in a previous proteomics studies we showed that AGAT protein expression in kidneys was significantly reduced when rats were exposed to sirolimus [19] suggesting that the differences in urine creatinine and urea concentrations observed in the present study may reflect differences in the effects of the study drugs on kidney metabolism.

It has to be taken into account that one problem with the targeted comparison of individual metabolites in selected treatments groups as shown in Table 2 and Figure 7 was that each of the treatment groups consisted of only n = 4 rats, offering low statistical power. However, with all the data taken into account our results showed a consistent pattern confirming the differences between EVL and SRL.

In addition, it is well established that oral bioavailability of the immunosuppressants tested exhibits rather large inter-individual variability and that doses often poorly correlate with blood concentrations. However, fairly good linearity between doses and drug concentrations (Figure 2) indicated the comparison of dose groups to be a valid approach. The differences between EVL and SRL on kidney metabolism were further confirmed by pooling the data of all dose groups and correlating blood concentrations with the effects on urine metabolite patterns (Figure 8). While with increasing blood concentrations of SRL concentrations of Krebs cycle metabolites in urine decreased, there was no corresponding blood-concentration-dependent decrease in the case of EVL.

The differences in SRL and EVL in combination with CsA as observed in the present study were also independently confirmed in a salt-depleted rat model to show that EVL in combination with CsA indeed leads to significantly less tubular injury than when corresponding doses of SRL and CsA were co-administered [51].

The molecular mechanisms underlying the differences between EVL and SRL observed in the present study require further investigation. It has been shown in a salt-depleted rat model that SRL enhanced CsA toxicity by promoting distribution of CsA into kidney tissue [40]. In normal fed rats, this was not the case with both EVL and SRL reducing kidney tissue concentrations of CsA to a similar extent in the present study (Table S1). Another potential explanation could have been one drug distributing more readily into the kidney tissue than the other. Therefore, we quantified SRL and EVL concentrations in the kidney. However, the results showed that EVL concentrations in the kidney were either similar or even higher than corresponding SRL concentrations (Tables S1C and S1D) rendering it unlikely that differences in tissue distribution were the cause for the observed differences. It was shown in perfused rat brain slices monitored real-time using wide-bore MRS that SRL enhances the negative effects of CsA by inhibiting anaerobic glycolysis, an alternative ATP-generating pathway that cells activated as a compensatory mechanism for the negative effects of CsA on mitochondrial oxidative phosphorylation [20]. In the same study, in contrast to SRL, EVL antagonized the negative effects of CsA on high-energy phosphate concentrations in perfused rat brain slices *via* an unknown mechanism [20]. It has been speculated that the differential effects on rat brain metabolism may be due to the fact that at therapeutically relevant concentrations EVL, but not SRL, can distribute into mitochondria [22]. If this is of relevance for the results observed in the present study remains to be seen.

As immunosuppressant nephrotoxicity is diagnosed based on histological changes and our study had the goal to assess changes in kidney biochemistry before or while only mild histological changes occurred, the question is if the differences on kidney metabolism observed in the present study will also translate into differences in nephrotoxicity. A large body of literature is available that has shown an association between changes in urine metabolite patterns and drug toxicity as confirmed by histological changes. Changes in urine metabolite patterns have extensively been used for the evaluation of kidney region specific toxins [52], [53], [54], [55], [56], [57], [58], [59], [60], [61], [62].

At this point, it is impossible to speculate if the results of the present study will have clinical implications. In the first phase III clinical trials with EVL, no difference to SRL in combination with full dose CsA was observed [15], [16], [63]. This is not surprising as previous studies had also indicated that SRL and EVL behave similar when CsA exposure is high with a CsA/EVL ratio of more than 5∶1 [22]. In the meantime, an increasing number of clinical studies have successfully used EVL in combination with low and ultra-low dose CNIs [64], [65], [66], [67], [68]. Overall, the present study clearly showed that in our rat model, although SRL and EVL are structurally related (Figure 1), their effects on urine metabolite patterns alone or in combination with CsA are markedly different with EVL having less of an effect on the rat kidney than SRL. This was especially the case when data were analyzed in context of the actual mTOR inhibitor blood and tissue concentrations rather than doses. It merits further exploration to study if our observations translate into a clinical benefit.

## Supporting Information

Table S1 **Kidney tissue concentrations of cyclosporine (CsA), sirolimus (SRL) and everolimus (EVL) if dosed alone or in combination for 28 days.** The numbers behind the drug names give the doses in mg/kg/d. Thus, for example, CsA 10.0/SRL 3.0 means that this group of rats was treated with a combination of 10 mg/kg/day cyclosporine and 3.0 mg/kg/day sirolimus for 28 days. Concentrations [ng/mg of tissue] are means± standard deviations (n = 4).
(DOCX)

Table S2 **Summary of individual histology injury scores.**
(DOCX)

Figure S1 **Composite and individual histology injury scores.** Kidney tissue samples for histology were collected after 28 days of treatment and 4 hours after the last dose. As described in the Methods section, injury scores were based on the levels of glomerulosclerosis, mesangial matrix expansion, isometric tubular vacuolization, tubular atrophy, interstitial fibrosis and arteriolar hyaline thickening according to Banff '97 recommendations [32]. Histology injury scores in all examined categories were added and individual data was plotted (Figure S1). The horizontal bars represent the median for each group. Please note that symbols may overlap and appear as one data point (n = 8 for controls, n = 4 for all other groups). The numbers in the x-axis labels give the doses in mg/kg/d. Thus, for example, CsA 10.0/SRL 3.0 means that this group of rats was treated with a combination of 10 mg/kg/day cyclosporine and 3.0 mg/kg/day sirolimus for 28 days.
(DOCX)

Figure S2 **Representative ^1^H-MRS spectra of urine extracts after treatment for 28 days (aliphatic region).** The total number of urine samples evaluated for each group was n = 4. Selected signal assignments: 1 lactate, 2 alanine, 3 acetate, 4 succinate, 5 2-oxoglutarate, 6 citrate, 7 dimethylamine, 8 trimethylamine, 9 dimethyl glycine, 10 creatine, 11a/b creatinine, 12 trimethylamine oxide, 13 taurine, 14 hippurate, 15 ß-glucose. The numbers behind the drug names are the doses in ng/mg/day.
(DOCX)

Figure S3 **Principal component analysis (PCA) of rat urine GC-MS metabolite spectra after 28 days of drug treatment (A) based on spectra recorded in urine from rats treated with 3 mg/kg/day EVL and 3 mg/kg/day SRL alone as well as the vehicle controls and from rats treated with combinations of CsA (10 mg/kg/day) with EVL or SRL (both 3 mg/kg/day).** The spectra were analyzed using a non-supervised PCA (Gene Spring MS, Agilent Technologies, Palo Alto, CA). The results confirmed the ^1^H-MRS data (see Figure 6B) that CsA (10 mg/kg/day)+ SRL (3 mg/kg/day) (CsA 10/SRL 3) had a different effect on urine metabolite patterns than CsA+EVL at the same doses (CsA 10/EVL 3). It was also interesting to note that CsA+EVL did not separate from the single drug treatments or the controls.
(DOCX)

Figure S4 **Representative GC-MS ion chromatogram of urine extracts after treatment for 28 days.** The representative ion chromatogram is from a rat treated with CsA (3 mg/kg/day). **Sample preparation:** For measurement of urine metabolites, 100 µL of urine was treated with 10 µL of urease 25 K unit/g at 37°C for 30 min. To extract the samples, 800 µL acetone was added into each sample in a glass tube provided with a Teflon-lined screw cap. The samples were shaken for 5 minutes and centrifuged at 13000 rpm for 10 min at 4°C.The upper phase was transferred into a centrifuge tube and dried down under nitrogen flow. To derivate the samples, 100 µL of N,O-bis (trimethylsilyl) trifluoroacetamide (BSTFA): pyridine 5∶1 v∶v were added into each vial and heated at 110°C for 70 minutes. Hereafter, the samples were cooled down at room temperature and transferred into HPLC vials containing a 200 µL insert. The samples were then measured by GC-MS. **GC-MS analysis:** GC-MS analysis was carried out using an Agilent Technologies 6890N Network gas chromatograph coupled to an Agilent Technologies 5973 Network quadropole mass selective detector. A DB-5ms (Agilent Technologies, Palo Alto, CA) capillary column (30 m×0.25 mm, 0.25 µM film thickness) was used for the separation of metabolites in urine. The temperature of the injector was 280°C, and the sample (1 µL) was injected in the splitless mode. The column temperature was set to 80°C, and ramped at 2°C/min to 130°C. Hereafter the temperature was ramped again at 3°C/min to 200°C and then ramped at a rate of 5°C/min to 280°C. Then the temperature was held at 320°C for 2 minutes. The mass selective detector was operated in the positive electron impact ionization scan mode.
(DOCX)
