# Supplementary material for: Everolimus and Sirolimus in Combination with Cyclosporine Have Different Effects on Renal Metabolism in the Rat
Source: PLoS One. 2012 Oct 31;7(10):e48063. doi: 10.1371/journal.pone.0048063 (PMC3485290; doi:10.1371/journal.pone.0048063)
Supplement: Table S1 — Kidney tissue concentrations of cyclosporine (CsA), sirolimus (SRL) and everolimus (EVL) if dosed alone or in combination for 28 days. The numbers behind the drug names give the doses in mg/kg/d. Thus, for example, CsA 10.0/SRL 3.0 means that this group of rats was treated with a combination of 10 mg/kg/day cyclosporine and 3.0 mg/kg/day sirolimus for 28 days. Concentrations [ng/mg of tissue] are means± standard deviations (n = 4). (DOCX) [file pone.0048063.s001.docx]

**Table S1.** *Kidney tissue concentrations of cyclosporine (CsA), sirolimus (SRL) and everolimus (EVL) if dosed alone or in combination for 28 days.* The numbers behind the drug names give the doses in mg/kg/d. Thus, for example, CsA 10.0/SRL 3.0 means that this group of rats was treated with a combination of 10 mg/kg/day cyclosporine and 3.0 mg/kg/day sirolimus for 28 days. Concentrations [ng/mg of tissue] are means± standard deviations (n=4).

1. **SRL concentrations alone and in combination with CsA**

|  | SRL 0.5 | SRL 1.5 | SRL 3.0 |
| --- | --- | --- | --- |
| alone | 0.6 ± 0.2 | 1.6 ± 0.4 | 1.5 ± 0.3 |
| CsA 3.0 | 0.7 ± 0.2 | 1.7 ± 0.2 | 2.7 ± 0.4 |
| CsA 6.0 | 2.6 ± 1.2 | 4.2 ± 0.8 | 4.4 ± 0.6 |
| CsA 10.0 | 3.1 ± 0.6 | 3.1 ± 0.6 | 5.0 ± 1.2 |

1. **EVL concentrations alone and in combination with CsA**

|  | EVL 0.5 | EVL 1.5 | EVL 3.0 |
| --- | --- | --- | --- |
| alone | 0.7 ± 0.1 | 1.5 ± 0.4 | 4.4 ± 0.3 |
| CsA 3.0 | 0.9 ± 0.1 | 3.7 ± 1.0 | 4.6 ± 0.3 |
| CsA 6.0 | 1.8 ± 0.4 | 4.0 ± 0.5 | 7.4 ± 2.1 |
| CsA 10.0 | 4.3 ± 2.8 | 6.2 ± 0.6 | 11.6 ± 0.6 |

1. **CsA concentrations alone and in combination with SRL**

|  | CsA 3.0 | CsA 6.0 | CsA 10.0 |
| --- | --- | --- | --- |
| alone | 5.1 ± 0.4 | 13.0 ± 3.6 | 20.9 ± 2.8 |
| SRL 0.5 | 3.9 ± 1.1 | 8.8 ± 0.3 | 13.1 ± 3.7 |
| SRL 1.5 | 3.7 ± 0.4 | 7.6 ± 1.3 | 14.1 ± 1.5 |
| SRL 3.0 | 3.9 ± 1.2 | 8.0 ± 2.0 | 13.3 ± 6.2 |

1. **CsA concentrations alone and in combination with EVL**

|  | CsA 3.0 | CsA 6.0 | CsA 10.0 |
| --- | --- | --- | --- |
| alone | 5.1 ± 0.4 | 13.0 ± 3.6 | 20.9 ± 2.8 |
| Evl 0.5 | 3.4 ± 1.5 | 8.0 ± 1.6 | 15.9 ± 2.4 |
| Evl 1.5 | 5.3 ± 0.8 | 12.3 ± 1.9 | 16.1 ± 1.5 |
| Evl 3.0 | 2.9 ± 0.8 | 13.9 ± 1.7 | 14.1 ± 4.4 |
