# Supplementary material for: Everolimus and Sirolimus in Combination with Cyclosporine Have Different Effects on Renal Metabolism in the Rat
Source: PLoS One. 2012 Oct 31;7(10):e48063. doi: 10.1371/journal.pone.0048063 (PMC3485290; doi:10.1371/journal.pone.0048063)
Supplement: Table S2 — Summary of individual histology injury scores. (DOCX) [file pone.0048063.s002.docx]

**Table S2.** *Summary of individual histology injury scores.*

| *mean scores*  n = 3,4 | | glomerulo-sclerosis | mesangial matrix  expansion | isometric tubular  vacuolization | tubular atrophy | interstitial fibrosis | arteriolar hyaline |
| --- | --- | --- | --- | --- | --- | --- | --- |
| vehicle | Median | 0 | 0 | 0 | 0 | 0 | 0 |
|  | Range | 0 | 0 | 0 | 0 | 0 | 0 |
| CsA 3 | Median | 0 | 0 | 1.5 | 0.5 | 0 | 0 |
|  | Range | 0 | 0 | 0 – 2 | 0 - 1 | 0 | 0 |
| CsA 6 | Median | 0 | 0 | 2 | 1 | 0 | 0 |
|  | Range | 0 | 0 | 1 – 2 | 1 - 1 | 0 | 0 |
| CsA 10 | Median | 0 | 0 | 1.5 | 1 | 0 | 0 |
|  | Range | 0 | 0 | 1 – 3 | 1 - 1 | 0 | 0 |
| Evl 0.5 | Median | 0 | 0 | 0 | 0.5 | 0 | 0 |
|  | Range | 0 | 0 | 0 – 1 | 0 - 1 | 0 | 0 |
| Evl 1.5 | Median | 0 | 0 | 0 | 0 | 0 | 0 |
|  | Range | 0 | 0 | 0 – 1 | 0 - 1 | 0 | 0 |
| Evl 3.0 | Median | 0 | 0 | 1 | 0.5 | 0 | 0 |
|  | Range | 0 | 0 | 0 – 2 | 0 - 1 | 0 | 0 |
| Srl 0.5 | Median | 0 | 0 | 0 | 0 | 0 | 0 |
|  | Range | 0 | 0 | 0 – 1 | 0 - 1 | 0 | 0 |
| Srl 1.5 | Median | 0 | 0 | 0 | 0 | 0 | 0 |
|  | Range | 0 | 0 | 0 – 1 | 0 - 1 | 0 | 0 |
| Srl 3.0 | Median | 0 | 0 | 0 | 0 | 0 | 0 |
|  | Range | 0 | 0 | 0 – 2 | 0 - 1 | 0 | 0 |
| CsA10/Evl0.5 | Median | 0 | 0 | 0.5 | 2 | 0 | 0 |
|  | Range | 0 | 0 | 0 – 1 | 1 - 3 | 0 - 1 | 0 |
| CsA10/Evl1.5 | Median | 0 | 0 | 1 | 2 | 0 | 0 |
|  | Range | 0 | 0 | 0 – 3 | 1 - 3 | 0 - 1 | 0 |
| CsA10/Evl3.0 | Median | 0 | 0 | 0 | 2 | 1 | 0 |
|  | Range | 0 | 0 | 0 – 2 | 2 - 3 | 0 - 1 | 0 |
| CsA10/Srl0.5 | Median | 0 | 0 | 0 | 1.5 | 0 | 0 |
|  | Range | 0 | 0 | 0 – 1 | 1 - 2 | 0 - 1 | 0 |
| CsA10/Srl1.5 | Median | 0 | 0 | 0.5 | 1.5 | 0 | 0 |
|  | Range | 0 | 0 | 0 – 1 | 2 - 3 | 0 - 1 | 0 |
| CsA10/Srl3.0 | Median | 0 | 0 | 0.5 | 2 | 0 | 0 |
|  | Range | 0 | 0 | 0 – 2 | 1 - 3 | 0 - 1 | 0 |
