# Supplementary material for: Everolimus and Sirolimus in Combination with Cyclosporine Have Different Effects on Renal Metabolism in the Rat
Source: PLoS One. 2012 Oct 31;7(10):e48063. doi: 10.1371/journal.pone.0048063 (PMC3485290; doi:10.1371/journal.pone.0048063)
Supplement: Figure S1 — Composite and individual histology injury scores. Kidney tissue samples for histology were collected after 28 days of treatment and 4 hours after the last dose. As described in the Methods section, injury scores were based on the levels of glomerulosclerosis, mesangial matrix expansion, isometric tubular vacuolization, tubular atrophy, interstitial fibrosis and arteriolar hyaline thickening according to Banff '97 recommendations [32]. Histology injury scores in all examined categories were added and individual data was plotted (Figure S1). The horizontal bars represent the median for each group. Please note that symbols may overlap and appear as one data point (n = 8 for controls, n = 4 for all other groups). The numbers in the x-axis labels give the doses in mg/kg/d. Thus, for example, CsA 10.0/SRL 3.0 means that this group of rats was treated with a combination of 10 mg/kg/day cyclosporine and 3.0 mg/kg/day sirolimus for 28 days. (DOCX) [file pone.0048063.s003.docx]

**Figure S1** *Composite* *and individual histology injury scores.* Kidney tissue samples for histology were collected after 28 days of treatment and 4 hours after the last dose. As described in the Methods section, injury scores were based on the levels of glomerulosclerosis, mesangial matrix expansion, isometric tubular vacuolization, tubular atrophy, interstitial fibrosis and arteriolar hyaline thickening according to Banff ’97 recommendations [32]. Histology injury scores in all examined categories were added and individual data was plotted (Figure S1). The horizontal bars represent the median for each group. Please note that symbols may overlap and appear as one data point (n=8 for controls, n=4 for all other groups). The numbers in the x-axis labels give the doses in mg/kg/d. Thus, for example, CsA 10.0/SRL 3.0 means that this group of rats was treated with a combination of 10 mg/kg/day cyclosporine and 3.0 mg/kg/day sirolimus for 28 days.
