# Supplementary material for: Everolimus and Sirolimus in Combination with Cyclosporine Have Different Effects on Renal Metabolism in the Rat
Source: PLoS One. 2012 Oct 31;7(10):e48063. doi: 10.1371/journal.pone.0048063 (PMC3485290; doi:10.1371/journal.pone.0048063)
Supplement: Figure S2 — Representative 1H-MRS spectra of urine extracts after treatment for 28 days (aliphatic region). The total number of urine samples evaluated for each group was n = 4. Selected signal assignments: 1 lactate, 2 alanine, 3 acetate, 4 succinate, 5 2-oxoglutarate, 6 citrate, 7 dimethylamine, 8 trimethylamine, 9 dimethyl glycine, 10 creatine, 11a/b creatinine, 12 trimethylamine oxide, 13 taurine, 14 hippurate, 15 ß-glucose. The numbers behind the drug names are the doses in ng/mg/day. (DOCX) [file pone.0048063.s004.docx]

**Figure S2.** *Representative ^1^H-MRS spectra of urine extracts after treatment for 28 days (aliphatic region).* The total number of urine samples evaluated for each group was n=4. Selected signal assignments: 1 lactate, 2 alanine, 3 acetate, 4 succinate, 5 2-oxoglutarate, 6 citrate, 7 dimethylamine, 8 trimethylamine, 9 dimethyl glycine, 10 creatine, 11a/b creatinine, 12 trimethylamine oxide, 13 taurine, 14 hippurate, 15 ß-glucose. The numbers behind the drug names are the doses in ng/mg/day.
