# Supplementary material for: Everolimus and Sirolimus in Combination with Cyclosporine Have Different Effects on Renal Metabolism in the Rat
Source: PLoS One. 2012 Oct 31;7(10):e48063. doi: 10.1371/journal.pone.0048063 (PMC3485290; doi:10.1371/journal.pone.0048063)
Supplement: Figure S3 — Principal component analysis (PCA) of rat urine GC-MS metabolite spectra after 28 days of drug treatment (A) based on spectra recorded in urine from rats treated with 3 mg/kg/day EVL and 3 mg/kg/day SRL alone as well as the vehicle controls and from rats treated with combinations of CsA (10 mg/kg/day) with EVL or SRL (both 3 mg/kg/day). The spectra were analyzed using a non-supervised PCA (Gene Spring MS, Agilent Technologies, Palo Alto, CA). The results confirmed the 1H-MRS data (see Figure 6B) that CsA (10 mg/kg/day)+ SRL (3 mg/kg/day) (CsA 10/SRL 3) had a different effect on urine metabolite patterns than CsA+EVL at the same doses (CsA 10/EVL 3). It was also interesting to note that CsA+EVL did not separate from the single drug treatments or the controls. (DOCX) [file pone.0048063.s005.docx]

**Figure S3.** *Principal component analysis (PCA) of rat urine GC-MS metabolite spectra after 28 days of drug treatment (A) based on spectra recorded in urine from rats treated with 3 mg/kg/day EVL and 3 mg/kg/day SRL alone as well as the vehicle controls and from rats treated with combinations of CsA (10 mg/kg/day) with EVL or SRL (both 3 mg/kg/day).* The spectra were analyzed using a non-supervised PCA (Gene Spring MS, Agilent Technologies, Palo Alto, CA). The results confirmed the ^1^H-MRS data (see Figure 6B) that CsA (10 mg/kg/day)+ SRL (3 mg/kg/day) (CsA 10 / SRL 3) had a different effect on urine metabolite patterns than CsA+EVL at the same doses (CsA 10 / EVL 3). It was also interesting to note that CsA+EVL did not separate from the single drug treatments or the controls.


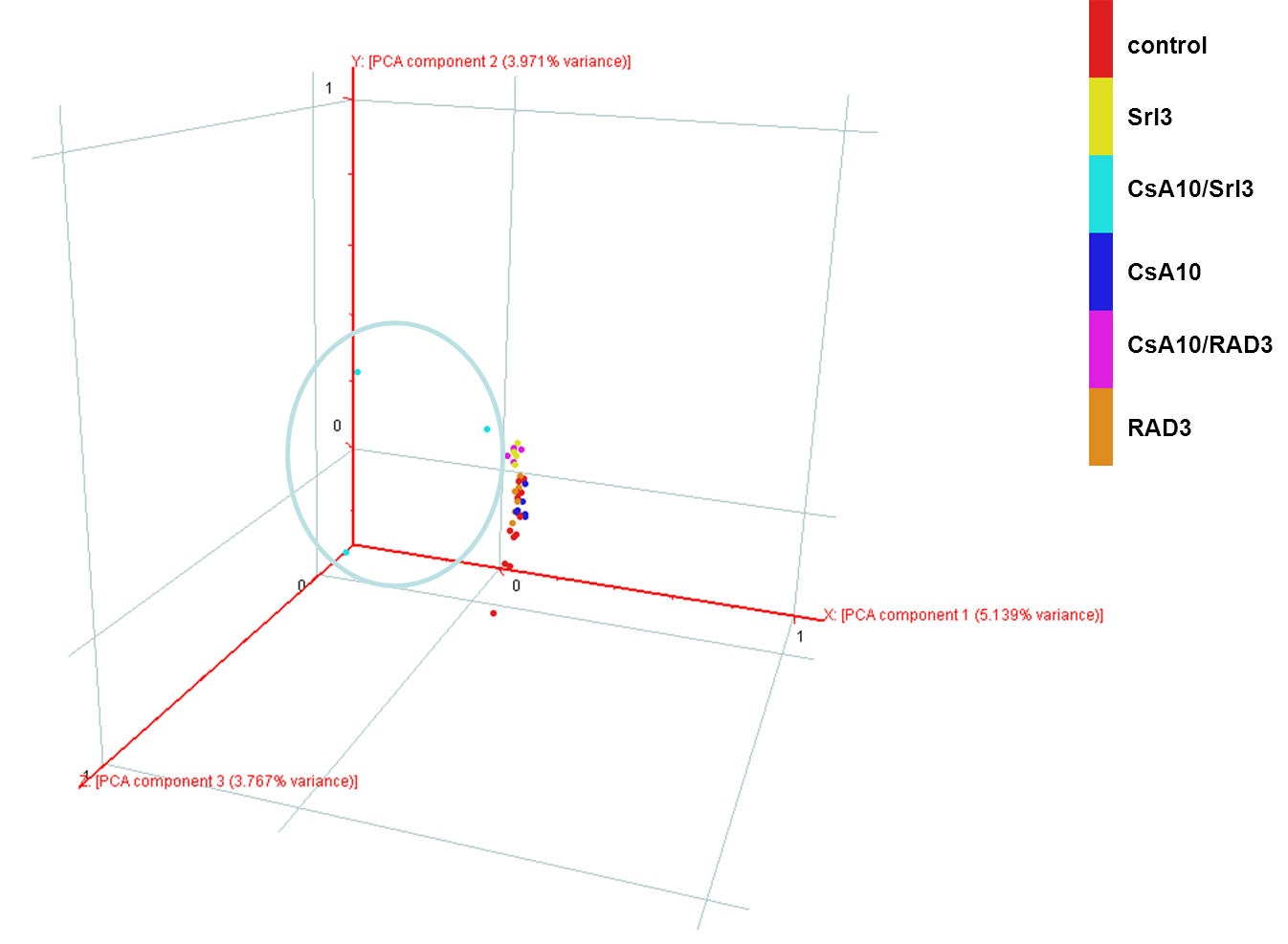


**CsA 10 / EVL 3**

**CsA 10**

**CsA 10 / SRL 3**

**SRL 3**

**Control**

**EVL 3**
