# Supplementary material for: Everolimus and Sirolimus in Combination with Cyclosporine Have Different Effects on Renal Metabolism in the Rat
Source: PLoS One. 2012 Oct 31;7(10):e48063. doi: 10.1371/journal.pone.0048063 (PMC3485290; doi:10.1371/journal.pone.0048063)
Supplement: Figure S4 — Representative GC-MS ion chromatogram of urine extracts after treatment for 28 days. The representative ion chromatogram is from a rat treated with CsA (3 mg/kg/day). Sample preparation: For measurement of urine metabolites, 100 µL of urine was treated with 10 µL of urease 25 K unit/g at 37°C for 30 min. To extract the samples, 800 µL acetone was added into each sample in a glass tube provided with a Teflon-lined screw cap. The samples were shaken for 5 minutes and centrifuged at 13000 rpm for 10 min at 4°C.The upper phase was transferred into a centrifuge tube and dried down under nitrogen flow. To derivate the samples, 100 µL of N,O-bis (trimethylsilyl) trifluoroacetamide (BSTFA): pyridine 5∶1 v∶v were added into each vial and heated at 110°C for 70 minutes. Hereafter, the samples were cooled down at room temperature and transferred into HPLC vials containing a 200 µL insert. The samples were then measured by GC-MS. GC-MS analysis: GC-MS analysis was carried out using an Agilent Technologies 6890N Network gas chromatograph coupled to an Agilent Technologies 5973 Network quadropole mass selective detector. A DB-5ms (Agilent Technologies, Palo Alto, CA) capillary column (30 m×0.25 mm, 0.25 µM film thickness) was used for the separation of metabolites in urine. The temperature of the injector was 280°C, and the sample (1 µL) was injected in the splitless mode. The column temperature was set to 80°C, and ramped at 2°C/min to 130°C. Hereafter the temperature was ramped again at 3°C/min to 200°C and then ramped at a rate of 5°C/min to 280°C. Then the temperature was held at 320°C for 2 minutes. The mass selective detector was operated in the positive electron impact ionization scan mode. (DOCX) [file pone.0048063.s006.docx]

**Figure S4.** *Representative GC-MS ion chromatogram of urine extracts after treatment for 28 days.* The representative ion chromatogram is from a rat treated with CsA (3 mg/kg/day). **Sample preparation:** For measurement of urine metabolites, 100 µL of urine was treated with 10 µL of urease 25K unit/g at 37°C for 30 min. To extract the samples, 800 µL acetone was added into each sample in a glass tube provided with a Teflon-lined screw cap. The samples were shaken for 5 minutes and centrifuged at 13000 rpm for 10 min at 4°C.The upper phase was transferred into a centrifuge tube and dried down under nitrogen flow. To derivate the samples, 100 µL of N,O-bis (trimethylsilyl) trifluoroacetamide (BSTFA): pyridine 5:1 v:v were added into each vial and heated at 110°C for 70 minutes. Hereafter, the samples were cooled down at room temperature and transferred into HPLC vials containing a 200 µL insert. The samples were then measured by GC-MS. **GC-MS analysis:** GC-MS analysis was carried out using an Agilent Technologies 6890N Network gas chromatograph coupled to an Agilent Technologies 5973 Network quadropole mass selective detector. A DB-5ms (Agilent Technologies, Palo Alto, CA) capillary column (30 m x 0.25 mm , 0.25 µM film thickness) was used for the separation of metabolites in urine. The temperature of the injector was 280°C, and the sample (1 µL) was injected in the splitless mode. The column temperature was set to 80°C, and ramped at 2°C /min to 130°C. Hereafter the temperature was ramped again at 3°C /min to 200°C and then ramped at a rate of 5°C /min to 280°C. Then the temperature was held at 320°C for 2 minutes. The mass selective detector was operated in the positive electron impact ionization scan mode.
